# Effect of a pharmacist‐led intervention on adherence among patients with a first‐time prescription for a cardiovascular medicine: a randomized controlled trial in Norwegian pharmacies

**DOI:** 10.1111/ijpp.12598

**Published:** 2019-12-29

**Authors:** Ragnar Hovland, Sara Bremer, Christine Frigaard, Solveig Henjum, Per Kristian Faksvåg, Erik Magnus Sæther, Ivar Sønbø Kristiansen

**Affiliations:** ^1^ Apokus, National Centre for Development of Pharmacy Practice Oslo Norway; ^2^ Norwegian Pharmacy Association Oslo Norway; ^3^ Oslo Economics Oslo Norway; ^4^ Department of Health Management and Health Economics University of Oslo Oslo Norway; ^5^ Present address: Pharmaq AS Oslo Norway; ^6^ Present address: Apotek 1 Inforama Sandvika Norway

**Keywords:** pharmacist‐led intervention, medication adherence, community pharmacy, hospital pharmacy, cardiovascular disease

## Abstract

**Objective:**

To examine whether a pharmacist‐led intervention improves medication adherence among patients who have filled a first‐time prescription for a cardiovascular medicine.

**Methods:**

Design: Unblinded randomized controlled trial**.** Setting: 67 Norwegian pharmacies, October 2014–June 2015. Participants: 1480 adults with a first‐time prescription for a cardiovascular medicine. Intervention: Participants in the intervention group received two consultations with a pharmacist 1–2 and 3–5 weeks after filling the prescription. Participants in the control group received care according to usual practice. Main outcome measure: The primary outcome was self‐reported adherence as measured by the 8‐item Morisky Medication Adherence Scale (MMAS‐8), at 7 and 18 weeks after filling the prescription. Adherence from baseline to week 52 was estimated using data from the Norwegian Prescription Database (NPD).

**Key Findings:**

Data from MMAS‐8 showed that 91.3% of the patients in the intervention group were adherent after 7 weeks versus 86.8% in the control group (4.5% difference, 95% CI 0.8–8.2, *P* = 0.017). The corresponding proportions were 88.7% versus 83.7% after 18 weeks (5.0% difference, 95% CI 0.8–9.2, *P* = 0.021). NPD data (*n* = 1294) showed no significant difference in adherence after 52 weeks (95% CI −2.0 to 7.8, *P* = 0.24). However, adherence among statin users (*n* = 182) was 66.5% in the intervention group versus 57.4% among new statin users in the general population (*n* = 1500) (difference 9.1%, 95% CI 1.5–16.0, *P* = 0.019).

**Conclusion:**

The main outcome measure indicates that a short, structured pharmacist‐led intervention may increase medication adherence for patients starting on chronic cardiovascular medication. However, these findings could not be confirmed by the NPD data analysis.

## Introduction

Poor adherence to prescribed medication for chronic diseases is a well‐documented and significant problem. In a report from 2003, the World Health Organization states that on average only 50% of patients adhere to their long‐term treatment.^1^ The implications of non‐adherence may be increased morbidity and mortality, and increased healthcare costs.^2^, ^3^, ^4^

Ischaemic heart disease and stroke are leading causes of death in Norway and most other countries.^5^, ^6^ Antihypertensive, cholesterol‐lowering and antithrombotic agents have been shown to be effective in the prevention of cardiovascular disease, but require good adherence to achieve their full benefit. In a meta‐analysis of 376 162 patients using seven different classes of cardiovascular drugs, the overall adherence was estimated to be 57% after 24 months.^7^

The reasons for non‐adherence are complex and diverse. It has been shown that higher adherence is associated with fewer concerns about the treatment, and a stronger perception of the necessity of the treatment.^8^ High cost, side effects and lack of effectiveness are commonly reported factors negatively influencing adherence.^9^ Other determinants of adherence are related to the drug treatment itself, including the type of drug and the complexity of treatment regimens.^1^ Many patients who experience problems leading to non‐adherence do not discuss them with the prescriber.

Interventions to improve medication adherence are often complex and time‐consuming.^10^ Adoption of such interventions into pharmacies is difficult. In addition, many interventions are mainly focused on patient education and are not necessarily addressing the true reasons for non‐adherence. Lastly, studies evaluating the effects of the interventions are often poorly designed, and the outcomes are inconsistent.^10^

Since 2011, accredited community pharmacies in England have been offering patients, who have been prescribed a new medicine for asthma, chronic obstructive pulmonary disease, hypertension, type 2 diabetes or an anticoagulant/antiplatelet agent, a pharmacist‐led intervention called the New Medicine Service (NMS). The intervention was developed taking into account that medication adherence is influenced by the patient’s unique symptoms or beliefs about the illness and the treatment.^11^, ^12^ It also acknowledges the fact that patients often experience problems and have substantial information needs in the first few weeks after starting a new medicine.^13^ The NMS was recently examined in a randomized controlled trial, showing an effect on patient adherence, along with reduced cost for the healthcare system.^14^, ^15^ Influenced by the NMS, a similar intervention named Medisinstart was developed in Norway. Medisinstart was first evaluated in 67 pharmacies in 2014–2015 (this study), before it was implemented as a publicly funded service in Norwegian pharmacies in 2018.

The primary aim of this study was to examine whether the Medisinstart intervention improved medication adherence among patients who have filled a first‐time prescription for a cardiovascular medicine, compared with usual pharmacy practice. Another aim was to investigate the impact of the intervention on the patients’ beliefs about medicines.

## Methods

### Trial design

The study was conducted as an unblinded, randomized controlled trial with two arms, comparing the pharmacist‐led intervention with usual pharmacy practice.

### Participants

The participants were recruited in 67 community and hospital pharmacies across Norway. The recruitment of pharmacies was performed by the pharmacy head offices and focused on including pharmacies with different sizes and locations. Participating pharmacies had to have at least two study pharmacists.

Patients 18 years and older, presenting themselves in one of the study pharmacies to collect a first‐time prescription (self‐report) for a study‐medicine (Table S1) for a cardiovascular condition, were asked to participate in the trial. Exclusion criteria were prior use of the medicine and a non‐cardiovascular indication. The participants signed a written informed consent.

### Randomization

After the pharmacist had dispensed the prescription, participants were randomly assigned (1:1) to either intervention or control. To ensure equal distribution of participants between the groups within the individual pharmacies and within the three therapeutic classes (anticoagulants, antihypertensive drugs and statins), a block‐randomization procedure was employed. In practice, the individual pharmacies randomized participants by drawing sealed envelopes from a cardboard box supplied by the research group. The box contained three groups of envelopes according to the class of study‐medicine. The envelopes contained a unique study ID and the randomization result. The order of the envelopes within each group was arranged by a random allocation sequence generated by the online tool, www.randomization.com.

### Intervention

Participants randomized to the intervention group received two consultations with a pharmacist, at 1–2 weeks and 3–5 weeks after filling their prescription for the first time. The participant decided whether the consultations should take place in the pharmacy, or over the phone. The allocated time for each consultation was 10–20 min.

A semi‐structured interview guide was used for the consultations (Appendix S1). The questions were based on the NMS interview schedule and designed to start conversations about correct use of the new medicine, what to do when doses were forgotten and how to prevent and relieve side effects. Furthermore, the guide was designed to address concerns and resolve misunderstandings. It was stressed that the information provided by the pharmacists should be individualized to the patients’ needs.

Registered pharmacists with minimum 6 months pharmacy practice were eligible to deliver the intervention after completion of a standardized training programme. The training involved a therapeutic update on the specific drug classes based on e‐learning courses and fact sheets. The training also involved an e‐learning course presenting the study and reading the study manual, interview guide and a list of standardized advice and information for patients. Finally, the pharmacists attended a full day practical training on how to deliver the intervention and adhere to the study protocol. More details are given in the training plan (Appendix S2). Further information will be available upon request.

The intervention and data collection processes were followed up by visiting the study pharmacies for a review of the study material and interviewing study pharmacists. Furthermore, the study pharmacies were followed up with regular phone calls.

### Control

Participants in the control group received standard care according to the pharmacy practice. Norwegian pharmacies have no routine follow‐up but offer advice on demand.

By using data from the Norwegian Prescription Database (NPD), 1500 individuals starting statin therapy during the period from July 2013 to June 2014 were identified as an additional, external control group representative of the general population. This control group was limited to statin users because of the simple dosing regimen and the size of this subpopulation.

### Outcomes

The primary outcome measure was self‐reported adherence to the new cardiovascular medicine, as measured by the 8‐item Morisky Medication Adherence Scale (MMAS‐8),^16^, ^17^, ^18^ at 7 and 18 weeks after filling the prescription. A validated Norwegian translation of the MMAS‐8 questionnaire was used. Participants with MMAS‐8 score ≥6 were classified as adherent.

A secondary self‐reported adherence outcome was measured by a slightly modified translation of the adherence question from the NMS.^15^, ^19^ A participant was considered adherent if, by self‐report, no doses were missed during the last seven days. This outcome was measured by questionnaire at 7 and 18 weeks after filling the prescription.

A third measure of adherence, Medication Possession Ratio (MPR), was measured for the 52 weeks period after filling of the first‐time prescription.^20^ The calculations were based on NPD data and dosing information obtained from the participants at inclusion. For the external control group of statin users, a dosage of one tablet daily was anticipated. For participants starting more than one new cardiovascular medicine at inclusion, the medicine for MPR calculation was prioritized as follows: anticoagulants > statins > antihypertensive drugs. The MPR was calculated as the number of daily doses dispensed for the period divided by 365 days. The dispensing in Norway is mainly based on whole packs of tablets, usually for 1–3 month’s supply. The calculation included corrections for changes in drug strengths and formulations that occurred before the expected time for the next refill. Furthermore, the calculations were also adjusted for switches between active ingredients within the same drug classes. Participants with an MPR ≥0.9 were considered adherent.

The secondary outcome measures concern and necessity were measured by a validated translation of the Beliefs about Medicines Questionnaire (BMQ) at baseline and 7 and 18 weeks after filling the first‐time prescription.^11^, ^21^ The necessity and concern subscales ranged from 5 to 25, where a high score reflects a strong belief (i.e. strong necessity or strong concern).

The baseline BMQ was filled out in the pharmacy before randomization. All other questionnaires, together with an instruction and a postage‐paid return envelope, were mailed to the participants by the study pharmacies 1–2 weeks before the date of filling it out. Notice was given (by phone or SMS) if the questionnaires were not received within one week after the deadline.

Licenses for using MMAS‐8 and BMQ were obtained.

### Subgroup analyses

Three subgroups were defined based on the type of new cardiovascular medicine: anticoagulants (ATC B01), antihypertensive drugs (ATC C07, C08 and C09) and statins (ATC C10).

### Statistical analysis

It was calculated that group sizes of 467 patients were needed to detect a difference in adherence of 7% for the primary outcome measure, using adherence incidences of 91% and 84% in the intervention‐ and control group, respectively (90% power, 5% significance). These incidences were based on the 4 weeks follow‐up results from a similar pharmacist‐led intervention.^12^ Allowing a 30% loss to follow‐up resulted in calculated group sizes of 667. It was therefore decided to aim at inclusion of 1500 participants.

Statistical analysis was performed using IBM® SPSS® Statistics (IBM Corporation, NY) version 25. For binary outcomes, the chi‐squared test was used for comparison between the arms. For continuous outcomes, the independent samples *t*‐test was employed. The Wilcoxon signed‐ranks test was used to compare the outcomes from the different self‐reported adherence measures (MMAS‐8 and adherence question). Differences are presented with 95% confidence intervals (CI). *P*‐values <0.05 were considered statistically significant. Values of continuous variables are presented as mean (standard deviation; SD), if not otherwise specified.

For all outcomes, the intention‐to‐treat principle was followed. All participants were mailed questionnaires and included in the data set used for MPR calculations regardless of whether they received the full intervention or not, or if the medicine was changed or discontinued during the trial. It was, however, decided not to impute missing data for the participants not answering the questionnaires, nor using data from the questionnaires completed outside of the predefined time‐period (5–9 weeks and 16–20 weeks).

### Ethical approval

The study was approved by the Regional Committee for Medical and Health Research Ethics on 5 May 2014 (2014/657).

## Results

**Figure 1:**
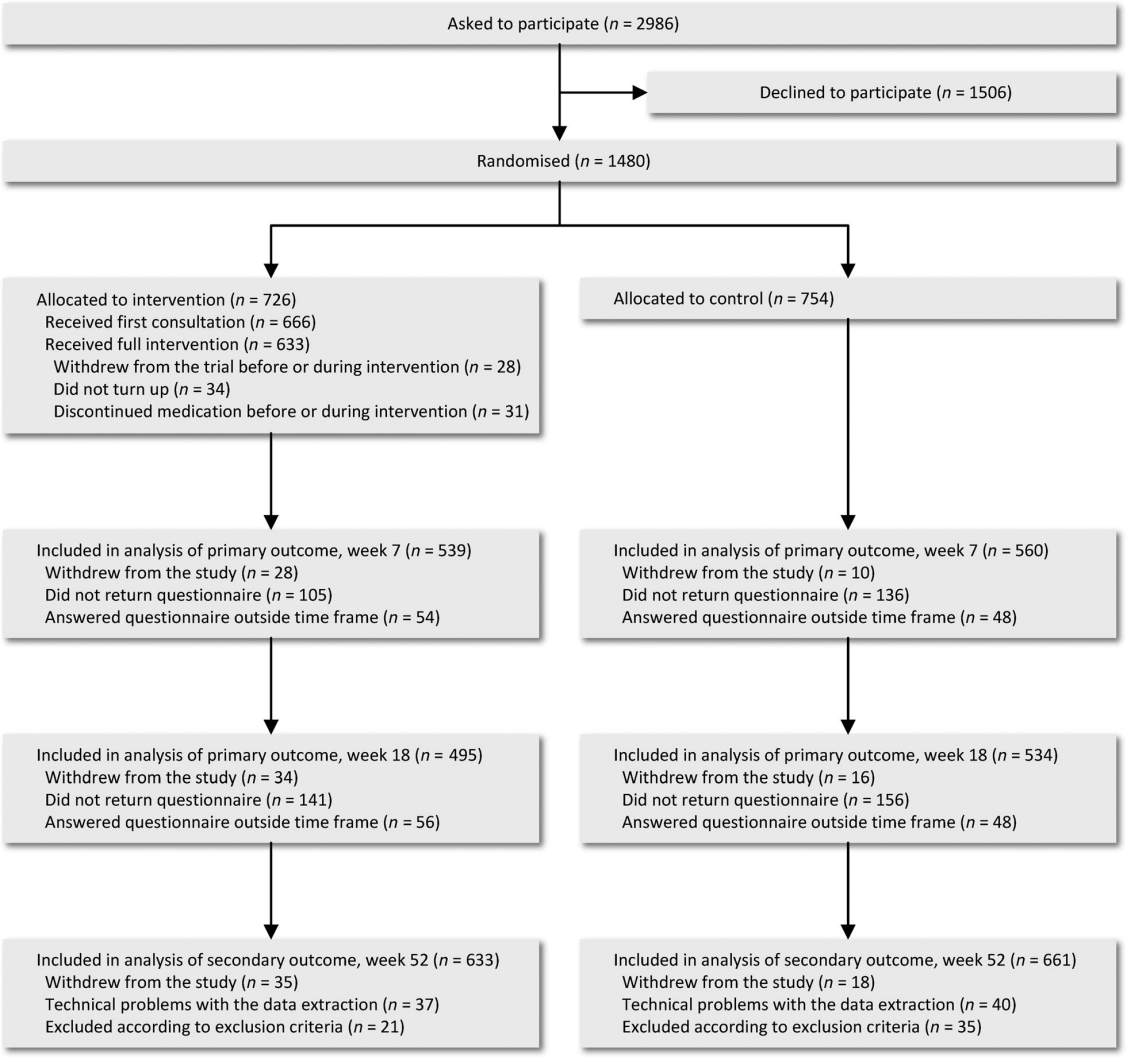
Flow diagram of the participants in the trial.

The CONSORT checklist summarizes the design, analyses and interpretation of the trial (Table S2). From October 2014 through June 2015, 1480 patients filling a first‐time prescription for a cardiovascular medicine were randomized to either the pharmacist‐led intervention or usual practice (Figure 1).

The 67 pharmacies participating in the trial represented all counties (*n* = 19) and 46 of the 428 (10.7%) municipalities in Norway. The number of pharmacy staff full‐time equivalents (FTEs)/pharmacy was 7.9 (4.0). For the hospital pharmacies, only FTEs working in the patient reception were counted. The mean number of FTEs for all Norwegian pharmacies in December 2015 was 8.1.^22^ The ownership of the pharmacies reflected the Norwegian pharmacy market, except for an overrepresentation of hospital pharmacies (Table S3). Each pharmacy recruited on average 22.1 (16.0) participants, ranging from 1 to 83.

Baseline characteristics were well balanced across the two groups and were representative of first‐time users of these drugs in Norway during the same period (Table 1). The frequencies of first‐time use of each active ingredient are presented in Table S1.

**Table 1:** Patient characteristics

Patient characteristics	Intervention	Control	Population*
Participants, *n* (%)	726 (49.1)	754 (50.9)	19 5417
Age (years), mean (SD)	62.0 (12.3)	61.9 (11.7)	n.a.
Men, *n* (%)	399 (55.0)	413 (54.8)	102 278 (52.3)
Number of new cardiovascular medicines, mean (SD)	1.17 (0.44)	1.16 (0.45)	n.a.
One new cardiovascular medicine, *n* (%)	619 (85.3)	655 (86.9)	n.a.
Two new cardiovascular medicines, *n* (%)	93 (12.8)	79 (10.5)	n.a.
Three new cardiovascular medicines, *n* (%)	12 (1.7)	18 (2.4)	n.a.
Four new cardiovascular medicines, *n* (%)	2 (0.3)	2 (0.3)	n.a.
Antithrombotic agents (B01), *n* (%)	109 (15.0)	114 (15.1)	19 381 (9.9)
Beta blocking agents (C07), *n* (%)	154 (21.2)	152 (20.2)	36 815 (18.8)
Calcium channel blockers (C08), *n* (%)	134 (18.5)	121 (16.0)	28 053 (14.4)
Renin–angiotensin system acting agents (C09), *n* (%)	229 (31.5)	257 (34.1)	63 371 (32.4)
HMG CoA reductase inhibitors (C10), *n* (%)	217 (29.9)	225 (29.8)	47 797 (24.5)
Number of additional medicines, mean (SD)	2.93 (2.48)	2.84 (2.65)	n.a.
First‐time cardiovascular medicine, *n* (%)	233 (32.1)	292 (38.7)	n.a.

* Total number of new users of the study‐medicines in Norway in the period 13 October 2014—19 June 2015. Data were obtained from the Norwegian Prescription Database. n.a., not available.

The time used for the intervention was 16.0 min (7.1) for the first consultation and 12.0 min (5.8) for the second consultation. The first and second consultations were carried out face‐to‐face with 414 (62%) and 347 (55%) of the participants, respectively. The remaining consultations were conducted by phone.

### Primary outcomes

Self‐reported adherence measured by MMAS‐8 at week 7 and week 18 is presented in Table 2. The proportion of adherent patients was 91.3% in the intervention group versus 86.8% in the control group after 7 weeks (*P* = 0.017), and 88.7% versus 83.7% after 18 weeks (*P* = 0.021). The response rates were 74.2% in both groups at 7 weeks, and 68.2% and 70.8% in the intervention and control group, respectively, after 18 weeks (Figure 1).

**Table 2:** Primary and secondary outcomes.

	Intervention	Control	Difference* (95% CI, *P*)
MMAS‐8	Number (%) of adherent patients		
Week 7 (*N* = 1099)	492 (91.3)	486 (86.8)	4.5% (0.8–8.2, **0.017**)
Week 18 (*N* = 1029)	439 (88.7)	447 (83.7)	5.0% (0.8‐9.2, **0.021**)
Adherence question	Number (%) of adherent patients		
Week 7 (*N* = 1098)	492 (92.0)	509 (90.4)	1.6% (−1.8 to 4.9, 0.36)
Week 18 (*N* = 1025)	456 (92.5)	468 (88.0)	4.5% (0.9–8.2, **0.015**)
Medication possession ratio	Number (%) of adherent patients		
Baseline to week 52 (*N* = 1294)	461 (72.8)	462 (69.9)	2.9% (−2.0 to 7.8, 0.24)
BMQ	Necessity score (SD)		
Baseline (*N* = 1454)	18.8 (3.9)	18.5 (4.0)	−0.3 (−0.7 to 0.1, 0.12)
Week 7 (*N* = 1088)	18.5 (3.8)	18.3 (3.7)	−0.2 (−0.7 to 0.2, 0.32)
Week 18 (*N* = 1014)	18.4 (3.6)	18.3 (3.7)	−0.1 (−0.5 to 0.4, 0.82)
Change, baseline to week 7 (*N* = 1075)	−0.3 (2.9)	−0.3 (3.2)	0.0 (−0.3 to 0.4, 0.84)
Change, baseline to week 18 (*N* = 1002)	−0.5 (3.2)	−0.3 (3.4)	0.2 (−0.2 to 0.6, 0.29)
BMQ	Concern score (SD)		
Baseline (*N* = 1454)	13.8 (3.9)	13.6 (4.1)	−0.2 (−0.6 to 0.2, 0.28)
Week 7 (*N* = 1082)	12.6 (3.8)	12.9 (3.9)	0.4 (−0.1 to 0.8, 0.12)
Week 18 (*N* = 1012)	12.4 (3.8)	12.6 (3.8)	0.2 (−0.3 to 0.7, 0.43)
Change, baseline to week 7 (*N* = 1071)	−1.4 (3.5)	−0.7 (3.6)	0.7 (0.3 to 1.1, **<0.001**)
Change, baseline to week 18 (*N* = 999)	−1.4 (3.5)	−1.0 (3.7)	0.5 (0.4 to 0.9, **0.034**)

MMAS‐8: 8‐item Morisky Medication Adherence Scale. The MMAS (8‐item) content, name, and trademarks are protected by US copyright and trademark laws. Permission for use of the scale and its coding is required. A license agreement is available from Donald E. Morisky, ScD, ScM, MSPH, MMAS Research LLC., 294 Lindura Ct. Las Vegas NV 89138‐4632, USA; dmorisky@gmail.com. BMQ, Beliefs about Medicines Questionnaire.

* Significant differences are shown with *P*‐values in bold.

### Secondary outcomes

The secondary outcomes are presented in Table 2. Self‐reported adherence measured by the adherence question was significantly higher in the intervention versus control group at 18 weeks (92.5% versus 88.0%, *P* = 0.015) but not at 7 weeks (92.0% versus 90.4%, *P* = 0.36). Adherence measured by the adherence question was higher than MMAS‐8‐based adherence at both time‐points (7 weeks: *Z* = 2.60, *P* = 0.009; 18 weeks: *Z* = 4.28, *P* < 0.001). Similar differences were observed when analysing the study groups separately, except for the intervention group where there was no difference between the measures at 7 weeks (*P* = 0.564).

According to MPR data, the adherence was 72.8% in the intervention group and 69.9% in the control group after 52 weeks (difference 2.9%, 95% CI −2.0 to 7.8, *P* = 0.24).

The intervention group demonstrated larger reductions in patients’ concern about the new medicine. The reductions in BMQ concern score after 7 weeks were 1.4 and 0.7 in the intervention and control groups, respectively (*P* < 0.001), while the corresponding reductions after 18 weeks were 1.4 and 1.0 (*P* = 0.034).

### Subgroup analyses

Adherence outcomes for the anticoagulant, antihypertensive and statin subgroups are presented in Table 3. The adherence among statin users was 91.0% in the intervention group versus 80.4% in the control group after 7 weeks (*P* = 0.007), and 87.9% versus 77.7% after 18 weeks (*P* = 0.022). In contrast, there were no significant differences between the intervention and control in the anticoagulant and antihypertensive subgroups.

**Table 3:** Adherence outcomes for the different groups of medicine

	Intervention	Control	Difference* (95% CI, p)
Anticoagulants	Number (%) of adherent patients		
Week 7 (MMAS‐8; *N* = 173)	77 (91.7)	83 (93.3)	−1.6% (−10.3 to 6.8, 0.69)
Week 18 (MMAS‐8; *N* = 162)	68 (94.4)	80 (88.9)	5.6% (−3.8 to 14.4, 0.21)
Baseline to week 52 (MPR; *N* = 200)	69 (69.7)	65 (64.4)	5.3% (−7.6 to 18.0, 0.42)
Antihypertensive drugs	Number (%) of adherent patients		
Week 7 (MMAS‐8; *N* = 723)	332 (91.5)	319 (88.6)	2.9% (−1.6 to 7.3, 0.20)
Week 18 (MMAS‐8; *N* = 683)	298 (88.2)	294 (85.2)	3.0% (−2.2 to 8.1, 0.26)
Baseline to week 52 (MPR; *N* = 715)	271 (77.0)	277 (76.3)	0.7% (−5.5 to 6.9, 0.83)
Statins	Number (%) of adherent patients		
Week 7 (MMAS‐8; *N* = 323)	141 (91.0)	135 (80.4)	10.6% (2.9–18.2, **0.007**)
Week 18 (MMAS‐8; *N* = 297)	123 (87.9)	122 (77.7)	10.2% (1.5–18.5, **0.022**)
Baseline to week 52 (MPR; *N* = 379)	121 (66.5)	120 (60.9)	5.6% (−4.1 to 15.1, 0.26)

MMAS‐8: 8‐item Morisky Medication Adherence Scale; MPR, Medication Possession Ratio.

* Significant differences are shown with *P*‐values in bold.

Comparing MPR‐based adherence for the intervention subgroup starting on statins (*n* = 182) with the external population‐based control group (*n* = 1500) demonstrated a 9.1% difference between the groups after 52 weeks (95% CI: 1.5–16.0; *P* = 0.019). The number of adherent patients in the external control group was 861 (57.4%). The difference in adherence between statin users in the control group (*n* = 197) and the external control group was 3.5% (95% CI: −3.9 to 10.5, *P* = 0.35). There were no significant differences between the groups with respect to patient age and indication for statin use (i.e. primary versus secondary prevention).

## Discussion

The results of the study showed that a pharmacist‐led intervention increased patients’ self‐reported adherence at 7 and 18 weeks after filling a first‐time prescription for a chronic cardiovascular medication. The increased adherence was accompanied by a decrease in patients’ concern about the new medicine. Subgroup analyses indicated that the intervention was particularly efficacious for patients starting on statins.

There were no significant differences in MPR‐based adherence at 52 weeks when analysing all drug classes combined. However, when comparing statin users with a population‐based control group, a difference in adherence was observed also at this time‐point.

### Strengths and limitations of the study

A major strength of the trial is that it was carried out in a real‐life setting in a selection of pharmacies largely reflecting the Norwegian pharmacy market. The intervention was carried out during regular working hours, as an integrated part of the pharmacists’ responsibilities.

The pragmatic study design also imposed some limitations. Baseline characteristics that could influence adherence, especially comorbidities (e.g. dementia and depression) and detailed information on concurrent medications are lacking. Lack of clinical outcome measures is another limitation of the study. Furthermore, the process evaluation was limited and not as thorough as recommended by the UK Medical Research Council.

Although the study was sufficiently powered for the primary outcome measure, even larger studies are needed to draw firm conclusions regarding subgroups and long‐term adherence.

For obvious reasons the trial was unblinded, for both the patient, pharmacist and analyst. The unblinded design might have led to differences in the dispensing service between groups. To decrease this risk, the patients were randomized after the prescription was dispensed. Furthermore, standardized information was given to all participants as part of the dispensing service. The standardized information contained one advice for each therapeutic group. The unblinded design also introduces a risk of differential questionnaire‐responses between the groups. This is especially relevant for self‐reported adherence. The moderate response rates may also have introduced a sampling bias.

Medication adherence is difficult to measure, and different methods have different limitations. Patients tend to overestimate their own adherence.^24^ A combination of adherence measures is therefore often used. In this study, self‐reported adherence was used as the primary outcome measure. A specific adherence question and MPR were used as additional adherence measures. Unfortunately, since 7 and 18 weeks are too short periods for meaningful use of MPR, no direct comparison with the self‐reported adherence measures was obtained.

### Comparison with other studies

Our findings are largely consistent with the findings from the evaluation of the NMS.^14^, ^15^ The NMS evaluation did not show any significant differences in self‐reported adherence between the groups at 6 weeks, but reported a 10.2% difference 10 weeks after filling the first‐time prescription. The evaluation also reported a good correlation between adherence as measured by MMAS‐8 and the adherence question used in the intervention. Our study showed significant differences in adherence measured by MMAS‐8 at both 7 and 18 weeks, while the adherence based on the adherence question only differed between groups at 18 weeks.

The adherence among patients in the NMS evaluation (control 60.5%, intervention 70.7% at 10 weeks) was lower than in our study. This can be explained by the fact that the NMS evaluation recruited patients that had been preselected for the intervention, either by general practitioners, the pharmacist or themselves, based on an anticipation of low adherence. In the current trial, all patients filling a prescription for a first‐time cardiovascular medicine were asked to participate. This probably also contributes to the somewhat larger effect observed in the NMS evaluation compared to the current trial. Other possible explanations could be the difference in medication classes between the studies and the different medicine reimbursement systems in England and Norway.

In the Pennsylvania Project, a brief pharmacist‐led intervention was offered to patients with elevated risk of non‐adherence using antihypertensive drugs, oral antidiabetic drugs or statins.^23^ The intervention was based on motivational interviewing and was carried out in community pharmacies across the United States.^24^ A randomized controlled trial with 59 496 patients showed statistically significant long‐term (270–365 days) effects on adherence for all medication classes investigated. The difference in adherence (measured as Percentage of Days Covered) between intervention and control was 4.1% (*P* < 0.001) for statin users. This is comparable to our observations of MPR‐based adherence after 52 weeks.

### Implication of findings and future research

Our results indicate that a pharmacist‐led intervention developed for Norwegian pharmacies increases adherence for patients starting with chronic cardiovascular medication. The increase in adherence is sustained for 18 weeks after filling the first‐time prescription and tends to last even longer among patients starting on statins.

There were no significant differences in MPR‐based adherence between the groups after 52 weeks. Interestingly, register data analysis showed that both the intervention and control group tended to have higher adherence than the general population. This may indicate an impact on the control patients that has masked the impact in the intervention group.

Further studies with larger and more homogenous patient groups are required to establish if the increased adherence will last over time, or if the intervention should be repeated at regular intervals. It has been shown by cost‐consequence analyses that a lasting increase in adherence by 1% is sufficient to make the intervention cost‐effective for new users of statins (Kristiansen IS and Sæther EM, unpublished data).

Future research should also examine the effect of the intervention by clinical and/or economic outcome measures. Given the generic nature of the intervention, it would be desirable to evaluate the effectiveness in other patient groups, or medication classes where adherence has been shown to be low.

## Conclusions

The results of the study indicate that a short, structured pharmacist‐led intervention, carried out in pharmacies as an integrated part of their services, may increase medication adherence for patients starting on chronic cardiovascular medication.

## Declarations

## Conflict of interest

The Author(s) declare(s) that they have no conflicts of interest to disclose.

### Funding

This work was supported by the Norwegian Pharmacy Association and the Norwegian Community Pharmacy Foundation.

### Acknowledgements

The authors would like to thank associate professor Matthew Boyd and professor Rachel Elliott at the University of Nottingham and Alastair Buxton, Director of NHS Services at the Pharmaceutical Services Negotiating Committee for sharing information, materials and experiences from the New Medicine Service (NMS). We would like to thank the patients who took part in the study and all involved pharmacies and pharmacy staff. Data from the Norwegian Prescription Database, the Norwegian Institute of Public Health (NIPH) were used for calculating MPRs. The MMAS (8‐item) content, name and trademarks are protected by US copyright and trademark laws. Permission for use of the scale and its coding is required. A license agreement is available from Donald E. Morisky, ScD, ScM, MSPH, MMAS Research LLC., 294 Lindura Ct. Las Vegas NV 89138‐4632, USA; dmorisky@gmail.com.

### Authors’ contributions

RH designed and implemented the trial, followed up the participating pharmacies, analysed the data and wrote the paper. SB analysed the data from the Norwegian Prescription Database. CF, PKF and SH designed and implemented the trial and followed up the participating pharmacies. EMS and ISK designed the trial. All authors revised and approved the final manuscript. All Authors state that they had complete access to the study data that support the publication.

## Supporting information


**Table S1**. Active ingredients included in the trial.

**Table S2**. CONSORT Statement 2010 Checklist. Items to include when reporting a randomized trial*.

**Table S3**. Pharmacies participating in the trial.

**Appendix S1**. Forms for follow‐up consultation 1 and 2.

**Appendix S2**. Training program for the Medisinstart study.
