# Supplementary material for: Effect of a pharmacist‐led intervention on adherence among patients with a first‐time prescription for a cardiovascular medicine: a randomized controlled trial in Norwegian pharmacies
Source: Int J Pharm Pract. 2019 Dec 29;28(4):337–45. doi: 10.1111/ijpp.12598 (PMC7384053; doi:10.1111/ijpp.12598)
Supplement: Supplementary file 1 — Table S1. Active ingredients included in the trial. [file IJPP-28-337-s001.docx]

Table S1: Active ingredients included in the trial

| ATC-group | Active ingredient | Number (%) of patients* | |
| --- | --- | --- | --- |
| B01 | Apixaban | 113 | (7.6) |
| B01 | Rivaroxaban | 96 | (6.5) |
| B01 | Dabigatran | 21 | (1.4) |
| C07 | Metoprolol | 246 | (16.6) |
| C07 | Bisoprolol | 31 | (2.1) |
| C07 | Carvedilol | 14 | (0.9) |
| C07 | Atenolol | 10 | (0.7) |
| C07 | Sotalol | 4 | (0.3) |
| C07 | Propranolol | 2 | (0.1) |
| C07 | Labetalol | 1 | (0.1) |
| C08 | Amlodipine | 138 | (9.3) |
| C08 | Nifedipine | 59 | (4.0) |
| C08 | Lercanidipine | 36 | (2.4) |
| C08 | Verapamil | 14 | (0.9) |
| C08 | Felodipine | 8 | (0.5) |
| C08 | Diltiazem | 1 | (0.1) |
| C08 | Isradipine | 0 | (0.0) |
| C09 | Candesartan | 145 | (9.8) |
| C09 | Losartan | 90 | (6.1) |
| C09 | Valsartan | 83 | (5.6) |
| C09 | Ramipril | 79 | (5.3) |
| C09 | Enalapril | 56 | (3.8) |
| C09 | Lisinopril | 17 | (1.1) |
| C09 | Irbesartan | 13 | (0.9) |
| C09 | Telmisartan | 3 | (0.2) |
| C09 | Olmesartan | 1 | (0.1) |
| C09 | Eprosartan | 0 | (0.0) |
| C09 | Captopril | 0 | (0.0) |
| C09 | Trandolapril | 0 | (0.0) |
| C10 | Atorvastatin | 305 | (20.6) |
| C10 | Simvastatin | 104 | (7.0) |
| C10 | Rosuvastatin | 23 | (1.6) |
| C10 | Pravastatin | 8 | (0.5) |
| C10 | Fluvastatin | 2 | (0.1) |
| C10 | Lovastatin | 1 | (0.1) |

*The percentages add up to more than 100% since some patients were included with more than one first-time prescription.
