# Supplementary material for: Effect of a pharmacist‐led intervention on adherence among patients with a first‐time prescription for a cardiovascular medicine: a randomized controlled trial in Norwegian pharmacies
Source: Int J Pharm Pract. 2019 Dec 29;28(4):337–45. doi: 10.1111/ijpp.12598 (PMC7384053; doi:10.1111/ijpp.12598)
Supplement: Supplementary file 2 — Table S2. CONSORT Statement 2010 Checklist. Items to include when reporting a randomized trial*. [file IJPP-28-337-s002.docx]

Table S2: CONSORT Statement 2010 Checklist. Items to include when reporting a randomized trial*

| **PAPER SECTION and topic** | **Item no.** | **Checklist item** | **Reported on page no. (section)** |
| --- | --- | --- | --- |
| **TITLE AND ABSTRACT** | | | |
|  | 1a | Identification as a randomized trial in the title | Page 1 (Title) |
|  | 1b | Structured summary of trial design, methods, results and conclusion | Page 4 (Abstract) |
| **INTRODUCTION** | | | |
| Background and objectives | 2a | Scientific background and explanation of rationale | Pages 6–7 (Introduction) |
|  | 2b | Specific objectives or hypotheses | Page 7 (Introduction) |
| **METHODS** | | | |
| Trial design | 3a | Description of trial design (such as parallel, fractional) including allocation ratio | Page 7 (Trial design), Page 8 (Randomization) and Figure 1 |
|  | 3b | Important changes to methods after trial commencement (such as eligibility criteria), with reasons | NA |
| Participants | 4a | Eligibility criteria for participants | Page 8 (Participants) |
|  | 4b | Settings and location where the data were collected | Page 8 (Participants) |
| Interventions | 5 | The interventions for each group with sufficient details to allow replication, including how and when they were actually administered | Page 8–9 (Intervention, Control), Appendix S1 and S2. |
| Outcomes | 6a | Completely defined pre-specified primary and secondary outcome measures, including how and when they were assessed | Page 10–11 (Outcomes) |
|  | 6b | Any changes to trial outcomes after the trial commenced, with reasons | NA |
| Sample size | 7a | How sample size was determined | Page 11 (Statistical analysis) |
|  | 7b | When applicable, explanation of any interim analyses and stopping guidelines | NA |
| Randomization – sequence generation | 8a | Method used to generate the random allocation sequence | Page 8 (Randomization) |
|  | 8b | Type of randomization; details of any restriction (such as blocking and block size) | Page 8 (Randomization) |
| Randomization – allocation concealment mechanism | 9 | Mechanism used to implement the random allocation sequence (such as sequentially numbered containers), describing any steps taken to conceal the sequence until interventions were assigned | Page 8 (Randomization) |
| Randomization - implementation | 10 | Who generated the random allocation sequence, who enrolled participants, and who assigned participants to interventions | Page 8 (Randomization) |
| Blinding | 11a | If done, who was blinded after assignment to interventions (for example, participants, care providers, those assessing outcomes) and how | NA |
|  | 11b | If relevant, description of the similarity of interventions | NA |
| Statistical methods | 12a | Statistical methods used to compare groups for primary and secondary outcomes | Pages 11–12 (Statistical analysis) |
|  | 12b | Methods for additional analyses, such as subgroup analyses and adjusted analyses | Pages 11–12 (Statistical analysis) |
| **RESULTS** | | | |
| Participant flow (a diagram strongly recommended) | 13a | For each group, the numbers of participants who were randomly assigned, received intended treatment, and were analyzed for the primary outcome | Figure 1 |
|  | 13b | For each group, losses and exclusions after randomization, together with reasons | Figure 1 |
| Recruitment | 14a | Dates defining the periods of recruitment and follow-up | Page 12 (Results) |
|  | 14b | Why the trial ended or was stopped | NA |
| Baseline data | 15 | A table showing baseline demographic and clinical characteristics for each group | Table 1 |
| Numbers analyzed | 16 | For each group, numbers of participants (denominator) included in each analysis and whether the analysis was by original assigned groups | Figure 1 and pages 11–12 (Statistical analysis) |
| Outcomes and estimation | 17a | For each primary and secondary outcome, results for each group, and the estimated effect size and its precision (such as 95% confidence interval) | Pages 12–14 (Results), Table 2 and 3 |
|  | 17b | For binary outcomes, presentation of both absolute and relative effect sizes is recommended | Table 2 and 3 |
| Ancillary analyses | 18 | Results of any other analyses performed, including subgroup analyses and adjusted analyses, distinguishing pre-specified from exploratory | Pages 12–14 (Results), Table 2 and 3. |
| Harms | 19 | All important harms or unintended effects in each group | NA |
| **DISCUSSION** | | | |
| Limitations | 20 | Trial limitations, addressing sources of potential bias, imprecision, and, if relevant, multiplicity of analyzes | Pages 14–15 (Strengths and limitations of the study) |
| Generalizability | 21 | Generalizability (external validity, applicability) of the trial findings | Pages 14–15 (Strengths and limitations of the study) |
| Interpretation | 22 | Interpretation consistent with results, balancing benefits and harms, and considering other relevant evidence | Pages 15–17 (Comparison with other studies, Implication of findings and future research) |
| **OTHER INFORMATION** | | | |
| Registration | 23 | Registration number and name of trial registry | Page 5 (Trial registration number) and page 12 (Ethical approval) |
| Protocol | 24 | Where the full trial protocol can be accessed, if available | NA |
| Funding | 25 | Sources of funding and other support (such as supply of drugs), role of funders | Pages 2–3 (Acknowledgements) |

*We strongly recommend reading this statement in conjunction with the CONSORT 2010 Explanation and Elaboration for important clarifications on all the items. If relevant, we also recommend reading CONSORT extensions for cluster randomized trials, non-inferiority and equivalence trials, non-pharmacological treatments, herbal interventions, and pragmatic trials. Additional extensions are forthcoming: for those and for up to date references relevant to this checklist, see www.consort-statement.org.
