# Supplementary material for: Effect of a pharmacist‐led intervention on adherence among patients with a first‐time prescription for a cardiovascular medicine: a randomized controlled trial in Norwegian pharmacies
Source: Int J Pharm Pract. 2019 Dec 29;28(4):337–45. doi: 10.1111/ijpp.12598 (PMC7384053; doi:10.1111/ijpp.12598)
Supplement: Supplementary file 3 — Table S3. Pharmacies participating in the trial. [file IJPP-28-337-s003.docx]

Table S3: Pharmacies participating in the trial.

| Pharmacy ownership | Number (%) of pharmacies in the trial | | Number (%) of pharmacies in Norway (December 2014) | |
| --- | --- | --- | --- | --- |
| Apotek 1 | 19 | (28.4) | 312 | (39.0) |
| Vitusapotek | 15 | (22.4)* | 214 | (26.8) |
| Hospital pharmacies | 15 | (22.4) | 32 | (4.0) |
| Boots apotek | 13 | (19.4) | 151 | (18.9) |
| Ditt apotek | 5 | (7.5)* | 73 | (9.1) |
| Independent pharmacies | 0 | (0.0) | 18 | (2.3) |

*One pharmacy changed ownership during the trial
