# Supplementary material for: Effect of a pharmacist‐led intervention on adherence among patients with a first‐time prescription for a cardiovascular medicine: a randomized controlled trial in Norwegian pharmacies
Source: Int J Pharm Pract. 2019 Dec 29;28(4):337–45. doi: 10.1111/ijpp.12598 (PMC7384053; doi:10.1111/ijpp.12598)
Supplement: Supplementary file 4 — Appendix S1. Forms for follow‐up consultation 1 and 2. [file IJPP-28-337-s004.pdf]

FORM FOR FOLLOW-UP CONSULTATION 1

ATTACH LABEL WITH STUDY ID HERE

Name:

Date:

Pharmacist:

Method (tick): ◯ BY PHONE ◯ AT THE PHARMACY

Time used:

min	min	min
PREPARATIONS	CONSULTATION	ADDITIONAL WORK

Summary of findings and advice/solutions recommended:

Status: ◯ SMS SENT ◯ 1 REMINDER ◯ 2 REMINDERS ◯ 3 REMINDERS ◯ ENDED

Medisin start

TURN THE PAGE →

Introduce yourself and briefly explain the purpose of the consultation + that the time frame for the consultation is 15 minutes.

Have you started using the new medicine? ◯ YES ◯ NO

- If NO → Can you tell me a little about what has prevented you?

Notes

How are you getting on with the new medicine?

Notes

How do you take your medicine during the day?

Notes

Which information have you received about why you are taking this medicine?

- Do you think the medicine is working? ◯ YES ◯ NO
- Has the effect been different from what you where expecting? ◯ YES ◯ NO
- Follow-up question → How?

Notes

Have you experienced any unexpected effects on the body after starting with the new medicine? ◯ YES ◯ NO

If YES → Which?

Notes

Do you have any concerns about using the new medicine? ◯ YES ◯ NO

If YES → Which?

Notes

People often miss taking a few doses of their medicines, for a wide range of reasons. Have you forgotten, or skipped, one or more doses of your new medicine? ◯ YES ◯ NO

- If YES → When did you last miss a dose?
- If FORGOTTEN → What do you think is the best solution to avoid forgetting to take the medicine?
- If SKIPPED → What do you consider is the most important reason for that?

Notes

Is there anything else you wish to know about your new drug, or is there anything else you would like us to talk about? ◯ YES ◯ NO

Notes

Summarize the consultation and actions you propose. Document these actions on the front of this form.

FORM FOR FOLLOW-UP CONSULTATION 2

ATTACH LABEL WITH STUDY ID HERE

Name:

Date:

Pharmacist:

Method (tick): ○ BY PHONE ○ AT THE PHARMACY

Time used:

min	min	min
PREPARATIONS	CONSULTATION	ADDITIONAL WORK

Summary of findings and advice/solutions recommended:

Status: ○ SMS SENT ○ 1 REMINDER ○ 2 REMINDERS ○ 3 REMINDERS ○ ENDED

Medisin start

TURN THE PAGE →

FOLLOW-UP CONSULTATION 2

WEEK 3-5

Introduce yourself and repeat the purpose of the consultation + that the time frame for the consultation is 15 minutes.

How have you been getting on with your new medicine since we last spoke?

- Are you still taking it? ○ YES ○ NO

Notes

How do you take your new medicine now?

Notes

Last time we spoke, you mentioned a few issues you'd been having. I'd like to follow these up with you today.

Last time you mentioned that (mention specific issue) **was a challenge. How is this now?**

- Did you try (refer to the solution proposed in follow-up interview 1) to alleviate this?
- What other solutions did you try?
- How did it help?

Notes

What other problems or concerns have you had since we last spoke?

Notes

People often miss taking a few doses of their medicines, for a wide range of reasons.
Have you forgotten, or skipped, one or more doses of your new medicine? ○ YES ○ NO

- If YES → When did you last skip a dose?
- If YES → How did you solve it?

Notes

Is there anything else you want us to talk more about? ○ YES ○ NO

Notes

Summarize the consultation and actions you propose. Document these actions on the front of this form. Inform the patient that he/she will be sent a questionnaire by mail within a few weeks.
