# Supplementary material for: Effect of a pharmacist‐led intervention on adherence among patients with a first‐time prescription for a cardiovascular medicine: a randomized controlled trial in Norwegian pharmacies
Source: Int J Pharm Pract. 2019 Dec 29;28(4):337–45. doi: 10.1111/ijpp.12598 (PMC7384053; doi:10.1111/ijpp.12598)
Supplement: Supplementary file 5 — Appendix S2. Training program for the Medisinstart study. [file IJPP-28-337-s005.pdf]

Medisin start

Training program for the Medisinstart study

Each study pharmacy must have at least two pharmacists (***Studiefarmasøyt/Study pharmacist***) competent to deliver follow-up consultations by using the Medisinstart interview-guides (the intervention). In addition, these pharmacists are responsible for implementing the study locally according to the procedures in the study manual, and making sure everyone involved are trained and adhere to the procedures in practice. Everyone (***Alle ansatte/ All employees***) dispensing prescriptions must be aware of the ongoing study, be able to provide standardized advice and information to the patient, be able to identify potential study participants and offer contact with a study pharmacist and be able to register those who decline to enter into the study. The pharmacy may have a need for pharmacists trained only to recruit participants to the study and make the first follow-up appointment if the study pharmacist is not available (***Inkluderingsfarmasøyt / Recruiting pharmacist***).

TRAINING PROGRAM FOR STUDY PHARMACISTS (STUDIEFARMASØYTER)

THERAPEUTIC UPDATE

In order to deliver the Medisinstart service (the intervention) at a high professional level it is necessary that the study pharmacist has updated knowledge and skills for all the drug classes included with regards to mode of action, correct use, adherence challenges, usual side-effects and how to manage these (e.g. prevention or relief).

E-learning courses

The E-learning courses listed below are recommended. Consider if there is a need for repetition if the courses have been completed previously (e.g. as part of the national pharmacy information campaign in 2013, NB2013). Each course has a time frame of approximately 30 minutes.

- HJ203 Antithrombotic prophylaxis part 1 (Tromboseprofylakse del 1)*
- HJ204 Antithrombotic prophylaxis part 2 (Tromboseprofylakse del 2)*
- HJ105 Antihypertensive treatment (Blodtrykksenkende behandling)
- HJ206 Antihypertensives and pharmacology (Antihypertensiva og farmakologi)
- HJ207 Hypertension and pharmacotherapy (Hypertensjon og farmakoterapi)
- HJ109 Cholesterol-lowering drugs (Kolesterolsenkende legemidler)

*Recommended literature about the new oral anticoagulants:
Informasjon om warfarin og de nye perorale antikoagulasjonsmidlene dabigatran, rivaroksaban og apixaban. Hdir, mars 2014. IS-2050.

Written material
Mandatory for all employees

- Standardized advice and information for patients (overview of advice/information that should always be given when dispensing medicines included in the study)

IMPLEMENTING THE MEDISINSTART STUDY

E-learning course
Mandatory for all study pharmacists, recommended for all employees

- AP102 The Medisinstart study (Medisinstart-studien). Time frame: approximately 20 minutes.

Written material
Mandatory for all study pharmacists

- Interview-guide 1: Follow-up consultation Week 1-2
- Interview-guide 2: Follow-up consultation Week 3-5
- Fact sheets for each drug class
- Medisinstart brochure
- Study manual (procedures on how to adhere to the study protocol)

1-day workshop with practical training

Mandatory for all study pharmacists

- Aim of Medisinstart (the intervention)
- Aim of the study, and overview on how to implement it in the pharmacy
- Communication skills
- Follow-up consultations
- Questions and answers

TRAINING PROGRAM FOR RECRUITING PHARMACISTS (INKLUDERINGSFARMASØYTER)

Recruiting pharmacists are to receive necessary training from a study pharmacist and know relevant procedures and the standardized advice and information for patients.

Mandatory:

- Selected study procedures
- Medisinstart patient brochure
- Standardized advice and information for patients

Training in the pharmacy

Recommended:

AP102 The Medisinstart study (Medisinstart-studien).

TRAINING PROGRAM FOR ALL EMPLOYEES (ALLE ANSATTE)

All employees are to receive necessary training from a study pharmacist and know relevant procedures and the standardized advice and information for patients.

Mandatory:

- Selected study procedures
- Standardized advice and information for patients

Training in the pharmacy

Recommended:

AP102 The Medisinstart study (Medisinstart-studien).
